# Non-invasive surface electroencephalography for broilers

**DOI:** 10.1016/j.mex.2025.103238

**Published:** 2025-02-19

**Authors:** Yukari Togami, Jolien Hacker, Elke Rauch, Michael Erhard, Helen Louton

**Affiliations:** a Chair of Animal Welfare, Ethology, Animal Hygiene and Animal Husbandry, Department of Veterinary Sciences, Faculty of Veterinary Medicine, LMU Munich, Veterinaerstr. 13/R, D-80539, Munich, Germany; b Animal Health and Animal Welfare, Faculty of Agricultural and Environmental Sciences, University of Rostock, Justus-von-Liebig-Weg 6b, D-18059, Rostock, Germany

**Keywords:** Electroencephalogram, Non-invasive-EEG, Surface electrodes, Broilers, welfare, Non-invasive surface electroencephalography for broilers

## Abstract

Our research aimed to develop a non-invasive EEG method for broiler chickens that can be utilized in basic research and practical applications. We evaluated the success of 1) the application and attachment of the EEG electrodes to the head of broiler and 2) measurements of EEG with non-invasive surface electrode (NISE). With this publication we would like to make the method NISE accessible to other researcher. We used a mobile EEG system with a combination of three surface electrodes, with a diameter of 8 mm. The mobile EEG device is carried by the broiler chickens in a backpack during the recording. Of the 52 broiler chickens examined using EEG, baseline data in a calm state was successfully recorded for 30 ss in 45 animals without significant artifacts. Measurement of EEG with a distance of 1 meter between the laptop and the EEG device in the backpack on the animal was successful, suggesting that this method could have broader applicability in practical environments in the future.

• The use of EEG measurements in veterinary research has grown.
• A non-invasive EEG technique for broiler chickens, suitable for both fundamental research and practical applications (NISE).
• A non-invasive method is more conducive to animal welfare.

## Graphical abstract

**Figure:**
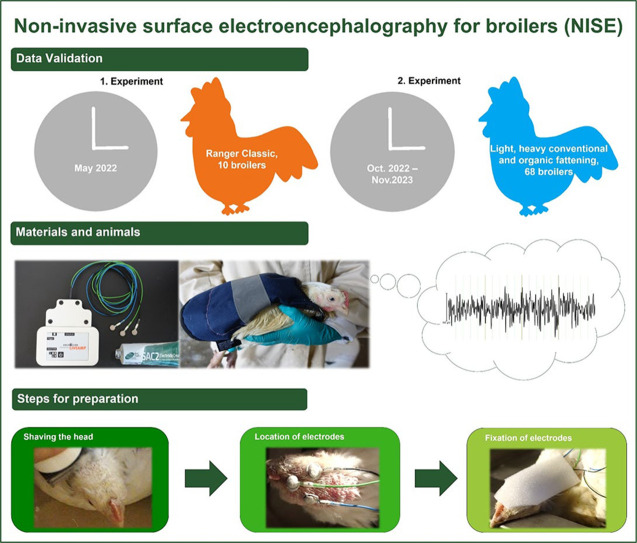

Specifications table

**Table:** 

Subject area:	Veterinary Science and Veterinary Medicine
More specific subject area:	*Animal welfare*
Name of your method:	*Non-invasive surface electroencephalography for broilers*
Name and reference of original method:	*N.A.*
Resource availability:	*N.A.*

## Background

The use of electroencephalography (EEG) in veterinary research has increased in recent years, particularly for assessing the effectiveness of stunning in animals. It has also been recommended as a direct, fast, and non-invasive method to monitor brain activity [1]. However, numerous of these studies, especially those involving chickens, predominantly employ invasive methods to reliably present data from moving animals without artifacts. Such approaches have so far only been feasible under controlled experimental conditions [2].

According to the EU Directive 2010/63/EU, animal experiments may only be conducted if their indispensability for advancing human knowledge and the corresponding harm to animals are unequivocally demonstrated (Article 38, Directive 2010/63/EU, 2010) [3].

Concurrently, less invasive methods are increasingly expected to be introduced as alternatives to animal experimentation from an animal welfare perspective, which either mitigate the burden on animals or eliminate the need of the use of animals in experiments. According to the worldwide standard 3R principle [4], the use of animals in experiments should be continuously reduced by developing and implementing methods that replace animals (Replace), reduce the number of animals needed (Reduce), and refine experimental conditions to minimize animal suffering (Refine). This presents a significant but necessary challenge for scientists, as the harmonization of animal welfare with the requirements of animal testing represents a complex and demanding interface.

In the specific context of bridging basic and applied research in the examination of animals, this study aims to develop a non-invasive EEG method for broiler chickens that can be applied in both fundamental research and practical settings. For this purpose, the success of 1) the application and attachment of the EEG electrodes to the chicken head and 2) the EEG measurement with non-invasive methods were tested. Once the EEG method has been established, this innovative method in the field of applied research will be made available to scientists in practical conditions, e.g. to determine the stunning effectiveness.

## Method details

### Animals

The research consisted of two experiments: the first, conducted at the experimental facility, focused on using non-invasive methods previously developed in the laboratory transferred to living animals, while the second, conducted at a commercial slaughterhouse, aimed to determine whether the realistic application of the method is feasible in a slaughterhouse with various environmental influences.

For the first part of the method development, sample tests were carried out on 10 female and male Ranger Classic broiler chickens (43 days old) with the experiment reference number (AZ): 7221.3–1–043/21 from 16.3.2022. These ten animals were examined in an experimental facility in May 2022. After this, the second experiment was then performed on a total of 68 female and male broiler chickens (between 30 and 63 days old) from October 2022 to November 2023 at a German commercial slaughterhouse (reference number: ROB-55.2–2532.Vet_02–21–80 from 16.03.2021, 05.08.2022 and 30.12.2022). The broilers used in the practical examinations were of the genotype Ross 308 for both light and heavy conventional fattening programs, and the Hubbard ISA JA 75 and 757 genotype for an organic fattening program. Animals of both examinations were randomly chosen shortly before the examination, ensuring a variety of genotypes and ages of broilers in the research.

## Materials and methods

### Non-invasive EEG method

**Fig. 1:**
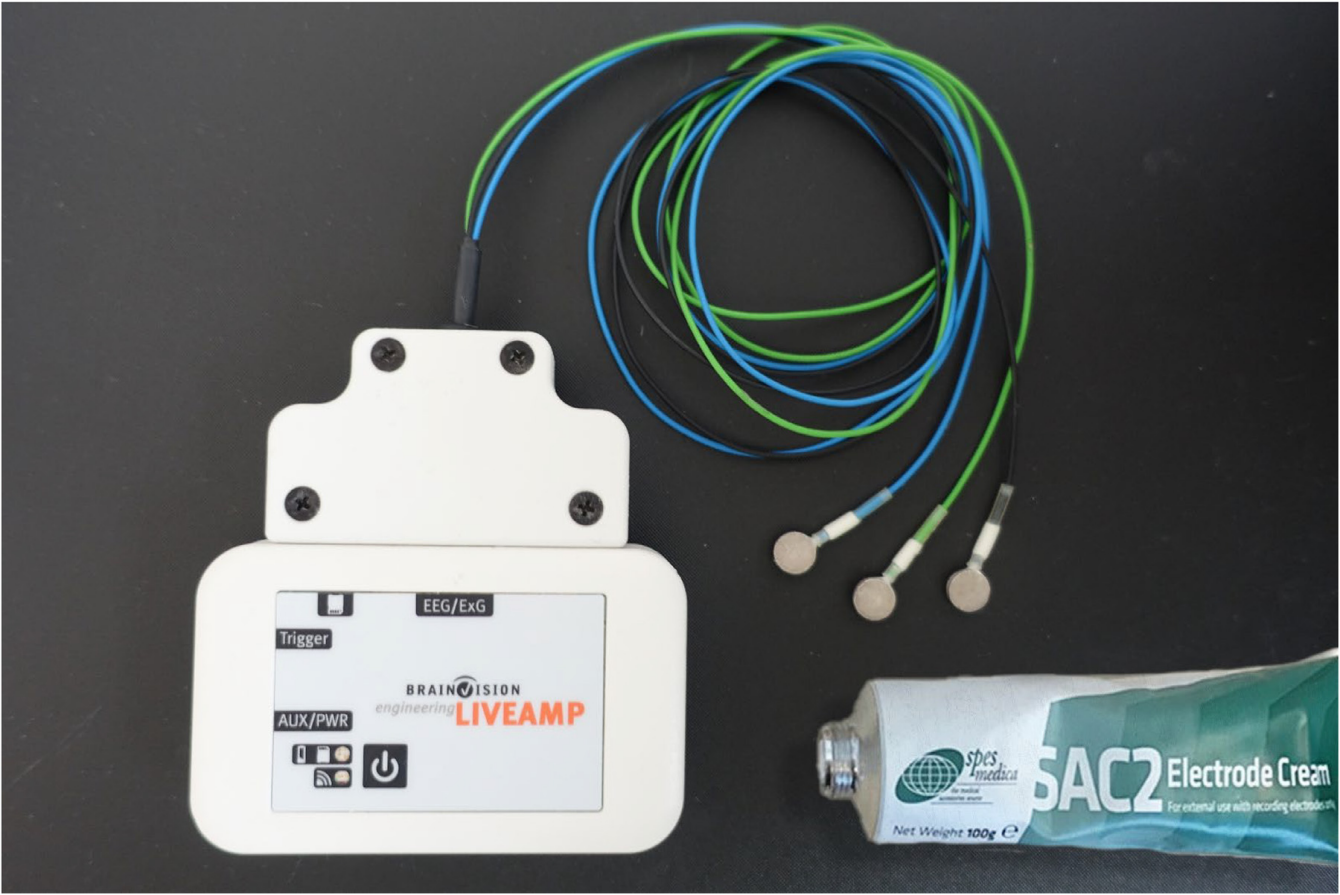
Mobile EEG (LiveAmp), Electrodes (8 mm diameter) and electrode creme (SAC2 Electrode Cream).

**Fig. 2:**
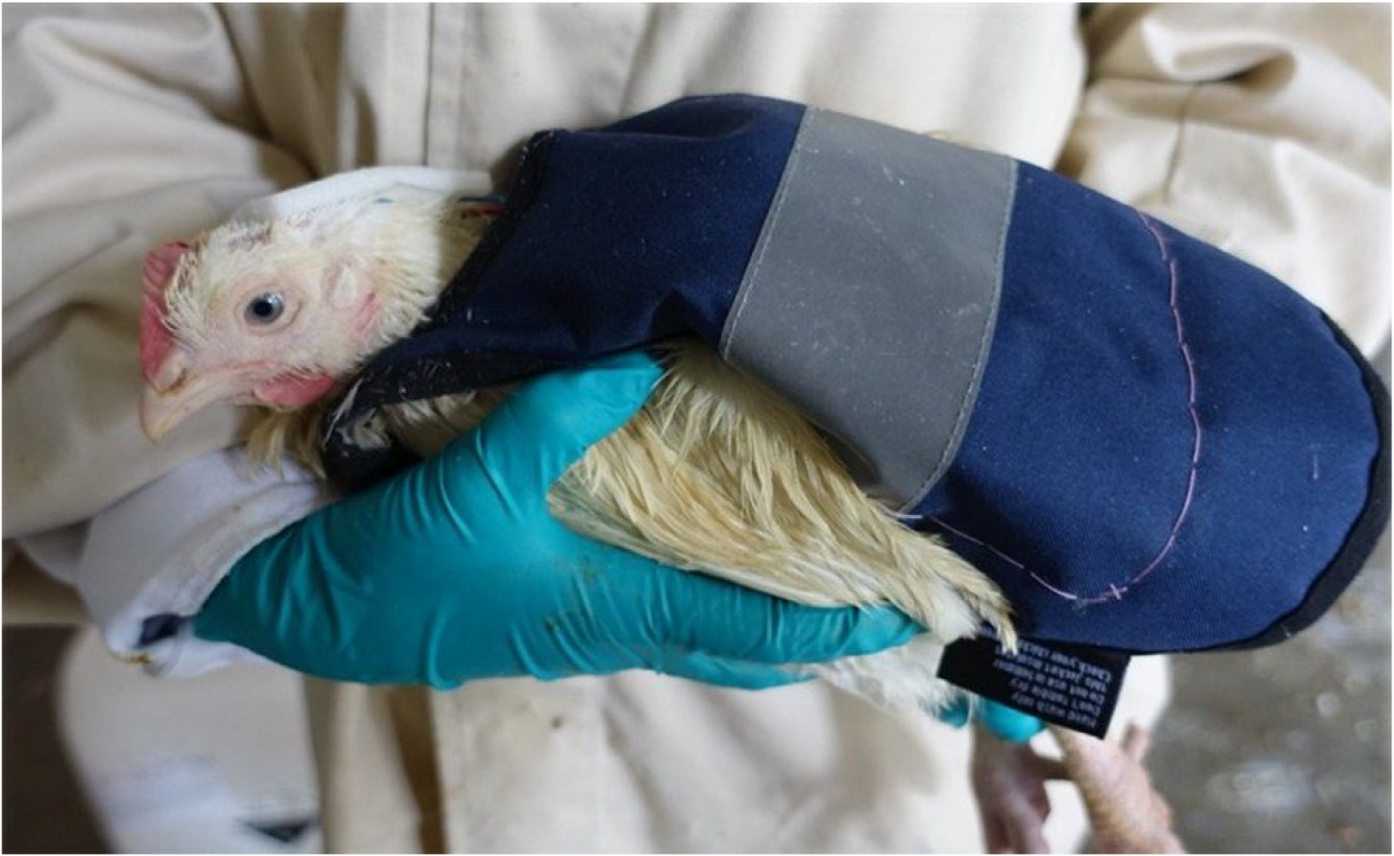
Broiler with mobile EEG in the backpack: Inside the backpack, there is a handmade, waterproof pocket for the mobile EEG unit. The EEG electrodes are attached to the head, sample animal: genotype Ross 308 of light conventional fattening program.

A mobile EEG system with passive surface electrodes (LiveAmp, BrainProducts GmbH, Gilching, Germany) was used to record the EEG signals (Fig. 1). A combination of three surface electrodes (a passive signal electrode and two additional electrodes: a reference electrode and a ground electrode), a diameter of 8 mm, with a mobile EEG system was developed during our research. The EEG-recording system (Brain Vision Recorder, BrainProducts GmbH, Gilching Germany) was connected to a laptop via a Bluetooth connection, and the mobile EEG device was carried by the chickens in a backpack during the EEG examination (Fig. 2).

### Attaching the electrodes to the head in 2 steps

The preparation for EEG recordings was performed in two steps:

**Step 1: Shave the head of the broiler**

**Fig. 3:**
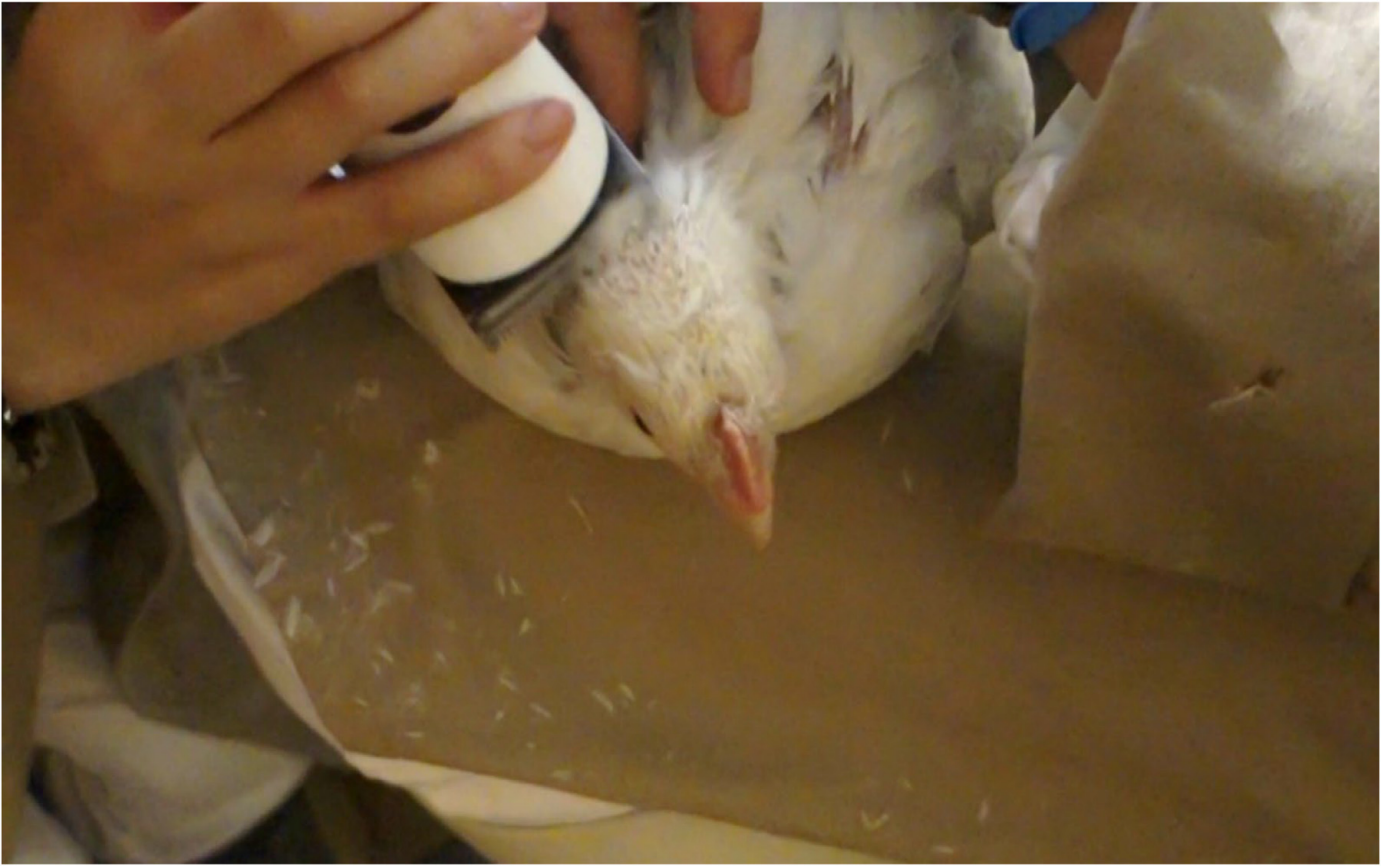
Shaving the head with a cordless clipper. The animals remained very calm during the shaving process, allowing the required area to be shaved within 30 ss, sample animal: genotype Ross 308 of light conventional fattening program.

First, the animal to be examined was carefully placed on a person's lap and held in place with both hands. After the animal had calmed down, the dorsal head was shaved directly behind the comb (Fig. 3). The heads of the chickens did not need to be held in place during shaving. The person shaving the head only placed a hand on the back of the neck to steady the head while shaving. This was done to create a clean and safe surface for the attachment of the EEG electrodes. A cordless clipper (Vega GT410 with clipper head GT606, Aesculap Schermaschinen GmbH, Suhl, Germany) was used for this purpose. A sound level meter (Decibel meter PCE-322A, PCE Instruments, Meschede, Germany) was used to carry out the measurement of noise of the clipper during the shaving in decibel (dB). The shaved area was then cleaned with a 70 % isopropyl alcohol swab.

**Step 2: Placement and fixation of EEG surface electrodes**

**Fig. 4:**
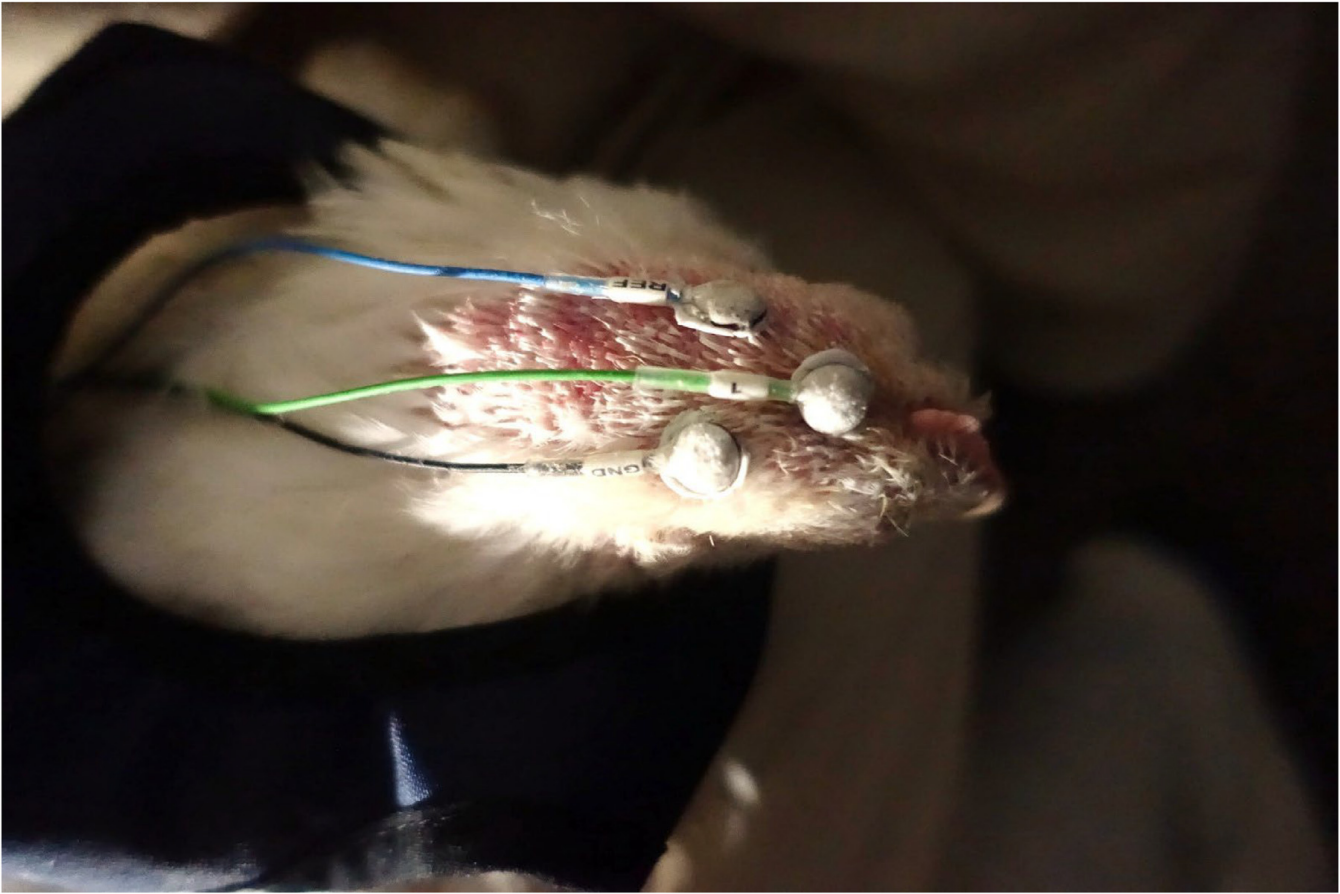
The localization of attached surface electrodes. An EEG adhesive paste was used between the electrodes and the scalp, sample animal: genotype Ross 308 of light conventional fattening program.

Three surface electrodes were placed on the shaved head. These electrodes were attached directly to the surface of the animal's head using a non-invasive (removable) EEG adhesive paste (SAC2 Electrode Cream, Spes Medica s.r.l. Genoa, Italy) to ensure proper electrode to skin contact (Fig. 4). All electrodes were placed approximately 0.5 cm behind the comb of the chickens to cover the brain activity of the cerebrum [5]. For additional fixation, the electrodes were covered with an adhesive bandage (Animal Polster, Snögg AS, Kristiansand, Norway) over the entire head from the comb to the neck.

### Measurement and analysis of brain activity

After the EEG-electrodes have been successfully attached to the chicken's head, brain activity was recorded in a quiet state for 30 ss (baseline EEG). “Quiet state" refers to the condition when the animals have calmed down after preparation for the EEG measurement and have shown no signs of defensive movements, such as head shaking or flapping, for 10 ss. Then we began measuring baseline EEG. During the EEG measurement, if the animals shook their heads or flapped their wings, we did not restrain them in order to observe whether the electrodes remained in place on the head. The movements of the animals were recorded three-dimensionally by a 3-axis acceleration sensor in the mobile EEG unit to exclude movement artifacts. The X, Y, and Z-axis data help detect and classify these artifacts, as the accelerometer is specifically suited for this purpose. Additionally, these three axes allow for a three-dimensional representation of the movements: The X-axis represents lateral (side-to-side) movements, the Y-axis corresponds to vertical (up-and-down) movements, and the Z-axis captures forward and backward movements. A notch filter at 50 Hz was applied to minimize interfering components of the mains frequency. Mains frequency refers to the frequency of the electrical power supply in a given region (e.g., 50 Hz in Europe). The recorded data were digitized at a sampling rate of 500 Hz and then processed with the BrainVision Analyzer 2.3 software (BrainProducts GmbH, Gilching, Germany) for evaluation. For our study, a digital band-pass filter was set with a lower cutoff at 1 Hz (low-cutoff, high-pass filter) and an upper cutoff at 40 Hz (high-cutoff, low-pass filter). Several studies have successfully conducted EEG recordings in birds using a sampling rate of >500 Hz [6,7]. This allows precise measurements and avoids aliasing effects, which is crucial when analyzing neural oscillations. In our study, we recorded EEG signals across the full frequency spectrum, without focusing on a specific band during the initial analysis. The primary goal was to ensure reliable EEG recordings under practical conditions. A detailed frequency-specific analysis of brain waves will be conducted in future studies . As a next step, we plan to apply Fast Fourier Transform (FFT) analysis to define and analyze specific frequency bands in more detail.

### Behavior measurement

A clear termination criterion for the experiment was established: the examination of an animal was stopped immediately if it exhibited two of the following behaviors: 1) a defensive reaction (such as wing flapping or vocalization) lasting longer than 10 ss, 2) a change in respiratory behavior e.g. an accelerated respiratory rate (over 30 breaths per minute) or a depressed respiratory rate (under 20 breaths per minute), 3) a pale head comb, or if the electrodes detached more than twice during preparation due to the animal shaking its head. To conduct EEG measurements in animals, the quiet state of the animals is a prerequisite. Since defensive reactions such as wing flapping or vocalization are often considered signs of stress in chickens for behavioral observation, it is also important to observe the behavior alongside the EEG measurement to determine when the measurement should be discontinued for animal welfare reasons.

## Method validation

The setup with the EEG device, Bluetooth connection, and backpack for the chickens is designed to enable simultaneous EEG monitoring for practical use by researchers, while also minimizing stress on the animals, maintaining a distance of approximately 1 meter between the animal and the examiner.

In the first experiment at the experimental facility, electrodes were smoothly placed on the heads of all 10 animals in <3 mins. However, the second experiment at the slaughterhouse revealed significant differences in animal behavior, leading to longer and more frequent preparation time. The sound pressure level of the clipper reached a maximum of 60 dB. The aim was to evaluate the impact of the clipper's loud noise on the chickens' behavior. The animals showed no stress reaction by the noise during shaving and we consider this method as reasonable. Noise levels above 80 dB are considered to induce significant stress in chickens, leading to physiological changes, increased stress hormone levels, and behavioral alterations [8]. However, due to environmental influences such as the traffic of transporters and forklift trucks or the alarm of the stunning system, the conditions in the slaughterhouse during the second experiment were not as quiet as in an experimental facility. The noise level in the slaughterhouse reached a maximum of 90 dB, allowing for a comparison between the general environmental noise and the noise of the clipper. A more detailed analysis of the specific impact of different noise sources on EEG recordings at various locations is needed as a next step.

These general conditions at the slaughterhouse caused restlessness among the animals and resulted in more movement in the second part of the experiment. Out of 68 animals, 16 often shook their heads, resulting in the experiment being aborted for those 16 animals before the EEG measurement could begin. Care should be taken, that the electrodes have to adhere to the head with the adhesive paste for at least one minute, and the experimenter has to hold them in place with light pressure. In the second experiment, attaching the electrodes took around 5 to 10 mins and sometimes had to be repeated, as the electrodes came off before the EEG was recorded and had to be reattached.

For both male and female broilers across all genetic lines, the 8 mm electrodes fit well. Localization of the electrodes 0.5 cm behind the comb is optimal, as placing them closer to the comb can result in artifacts, such as spindle-like activity [9], from eye movements.

**Fig. 5:**
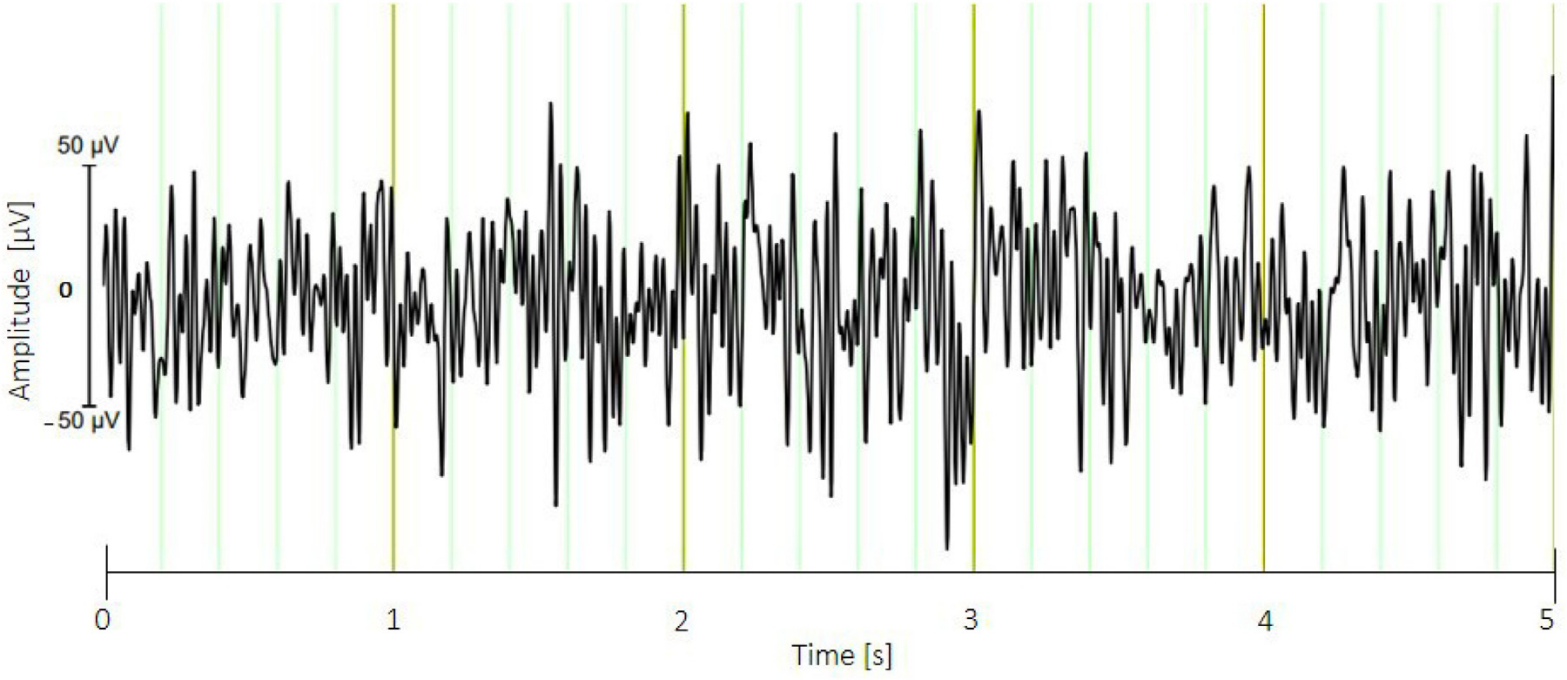
Baseline EEG without artifacts. Vertical line (y-axis) shows the amplitude of signal: Range 100 µV (Min: −50 µV, Max: 50 µV, Horizontal line (x-axis) shows the time in seconds, sample animal: genotype Ross 308 of light conventional fattening program.

**Fig. 6:**
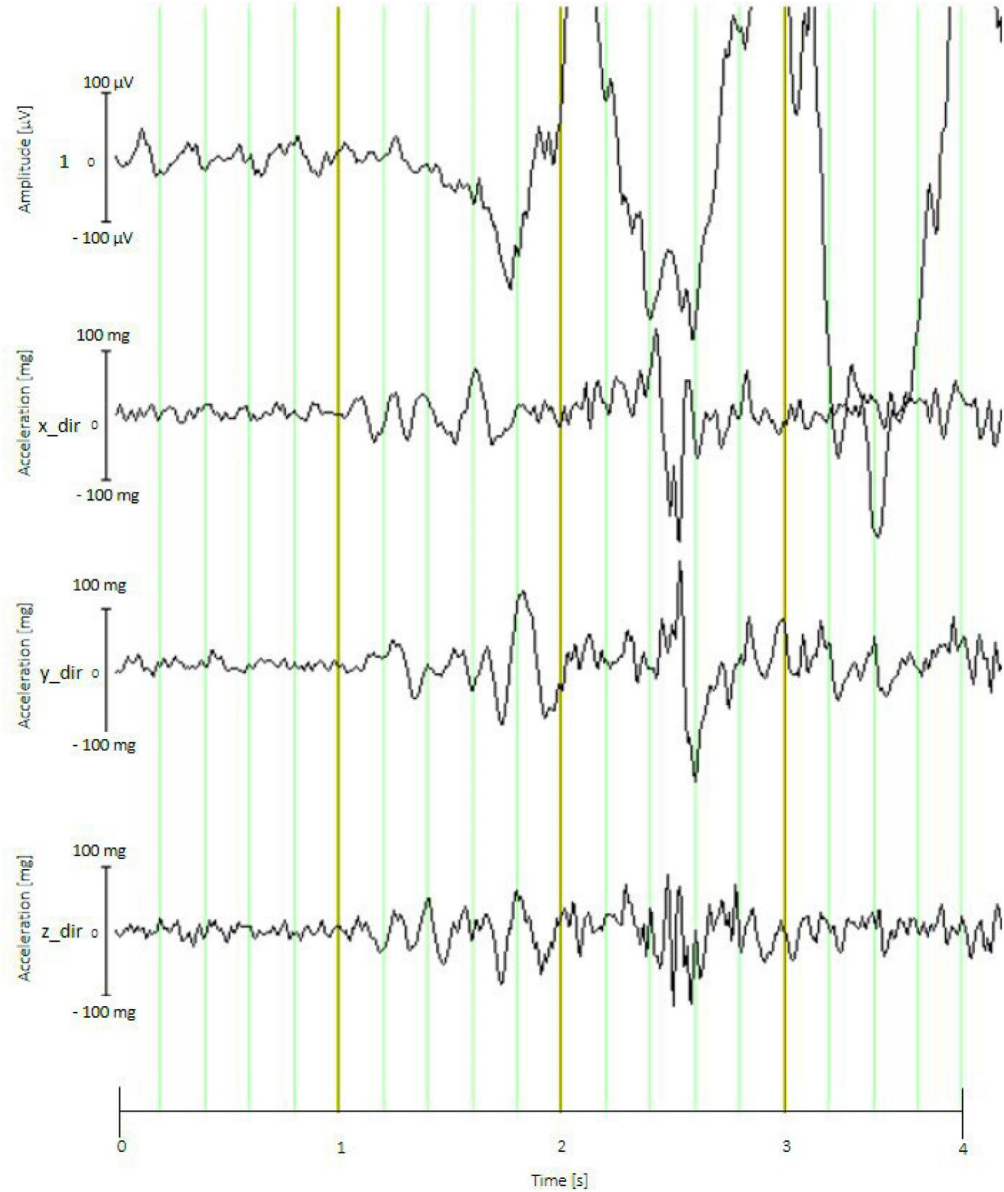
The top line (1) shows the EEG recording from the active electrode with movement artifacts from the second box (Vertical line (y-axis) shows the amplitude of signal: Range 200 µV (Min: −100 µV, Max: 100 µV, Horizontal line (x-axis) shows the time in seconds, while the lower three lines (x_dir, y_dir, and z_dir) correspond to head and body movements (simultaneous recordings) detected by the 3-axis acceleration sensor: Range 200 mg = milli gravity (Min: −100 mg, Max: 100 mg), sample animal: genotype Ross 308 of light conventional fattening program.

Out of the total of 52 broiler chickens examined with EEG measurements, baseline EEG data could be recorded for 30 ss from 45 animals without significant artifacts. Significant artifacts were considered as such if the electrodes did not remain stable on the chicken's head, making EEG recording impossible. For example, as soon as the experimenter left the animals alone, some chicken began to shake their heads repeatedly, causing the electrodes to come off or resulting in strong movement artifacts in the EEG data. This was observed in 7 animals before the baseline measurements could be completed (Fig. 5, Fig. 6), resulting in the 45 animals without artifacts. The use of an accelerometer in the mobile EEG was one method employed to identify movement artifacts caused by the animals' repetitive movements. The EEG measurement with a distance of 1 meter between the laptop and the EEG device (on the animal) was successful, suggesting that this method could have broader applicability in practical environments in the future, for example for the use in a barn. This research demonstrates the feasibility of non-invasive EEG examinations in broiler chickens under practical conditions. The results showed that non-invasive EEG recording in broiler chickens is feasible, preferably under low-stress conditions. The success rate of measurements will increase if the experimenters are nearby and can quickly intervene if the animals shake their heads. Many practical places, such as chicken farms or slaughterhouses, where EEG examinations might be conducted in the future, do not provide a lab-like environment for the animals or for the EEG measurements.

In general, EEG measurements require a preferably calm and stable environment. In our study, we demonstrated for the first time that non-invasive EEG recordings in chickens are possible under practical conditions without the need for invasive electrodes, and we examined how the animals respond to the preparation process—for example, shaving the head in a noisy environment after transportation from the farm. EEG recordings under such conditions had not been previously investigated, making this study a significant challenge. It is possible to calm the animals in practical settings and conduct EEG measurements, even though the conditions are not 100 % identical to those without human handling. However, this represents a major step forward, as it enables EEG recordings in farm animals under practical conditions without invasive methods. The main objective of this initial study was to develop and validate the non-invasive method itself.

Such EEG measurements will be highly valuable for future animal welfare assessments. As researchers, we often rely on potentially biased behavioral observations to assess animal well-being. Physiological parameters such as heart rate—or even EEG—offer a more objective and scientifically robust way to determine the animals' true state, which can be effectively used to promote positive welfare. We therefore would like to make this non-invasive EEG measurement method accessible to other researchers and further develop it into a truly practical and applicable tool for several conditions.

## Limitations

Greater emphasis should be placed on enhancing the stability of the electrode adhesive after shaving, to minimize the need for frequent adjustments and handling of the animals during measurements. It is crucial to advance methods that allow for an improved understanding of the animals through less invasive approaches. A common issue is the instability of the electrode adhesive, which is not due to normal, minimal animal movements.

While the electrode design includes a reference electrode, and differential recording can help reduce movement artifacts, some artifacts may still occur due to the continuous movement of the animals. Completely “clean” data are difficult to achieve under practical conditions. Although these artifacts can be minimized, they cannot be completely eliminated. However, we aimed to minimize these artifacts as much as possible during data processing. In this study, we used a higher sampling rate of 500 Hz. For future use of non-invasive EEG recording, a sampling rate of 250 Hz or lower may be considered to help reduce environmental noise while still capturing the relevant EEG signals. Under practical conditions, environmental factors such as transporter and forklift traffic or alarms from the stunning system, meant that the test environment was not as quiet as in an experimental facility. Nevertheless, our investigation showed that such factors do not cause significant signal artifacts in the EEG recordings, such as recording errors.

## Ethics statements

This research involving animal experiments complied with the ARRIVE guidelines and was conducted in accordance with EU Directive 2010/63/EU for animal experiments. All procedures adhered to ethical standards for the care and use of animals, as outlined in the National Institutes of Health guide for the care and use of laboratory animals (NIH Publications No 8023, revised 1978). The animals used in the research were of both sexes, and the potential influence of sex on the research's results was carefully considered in the research.

## Funding

The project was supported by funds of the 10.13039/501100005908Federal Ministry of Food and Agriculture (BMEL) based on a decision of the Parliament of the Federal Republic of Germany via the Federal Office for Agriculture and Food (BLE) under the innovation support program (Grant Number 2817804A18, Project CasStunn).

The authors extend their gratitude to all workers at the slaughterhouse for their invaluable assistance and support during the CasStunn project.

## CRediT authorship contribution statement

**Yukari Togami:** Methodology, Validation, Investigation, Formal analysis, Data curation, Writing – original draft. **Jolien Hacker:** Investigation, Writing – review & editing. **Elke Rauch:** Conceptualization, Writing – review & editing, Funding acquisition. **Michael Erhard:** Conceptualization, Supervision, Funding acquisition. **Helen Louton:** Conceptualization, Writing – original draft, Project administration, Funding acquisition.

## Declaration of competing interest

The authors declare that they have no known competing financial interests or personal relationships that could have appeared to influence the work reported in this paper.

## Data Availability

The data that has been used is confidential.
